# Biocontrol Potential of Rhizospheric Bacillus Strains Against *Sclerotinia minor* Jagger Causing Lettuce Drop

**DOI:** 10.3390/microorganisms13010068

**Published:** 2025-01-02

**Authors:** Lihui Xu, Qinghua Shang, Mogens Nicolaisen, Rong Zeng, Shigang Gao, Ping Gao, Zhiwei Song, Fuming Dai, Jingze Zhang

**Affiliations:** 1 Institute of Eco-Environmental Protection, Shanghai Academy of Agricultural Sciences, Shanghai 201403, China; xulihui@saas.sh.cn (L.X.); zengrong@saas.sh.cn (R.Z.); gaoshigang@saas.sh.cn (S.G.); gaoping@saas.sh.cn (P.G.); songzhiwei@saas.sh.cn (Z.S.); 2 Shanghai Key Laboratory of Protected Horticultural Technology, Shanghai 201403, China; 3 Shanghai Engineering Research Centre of Low-carbon Agriculture (SERCLA), Shanghai 201415, China; 4 State Key Laboratory of Rice Biology and Breeding, Ministry of Agriculture Key Laboratory of Molecular Biology of Crop Pathogens and Insects, Institute of Biotechnology, Zhejiang University, Hangzhou 310058, China; qhshang@zju.edu.cn; 5 Department of Agroecology, Faculty of Technical Sciences, Aarhus University, 4200 Slagelse, Denmark; mn@agro.au.dk

**Keywords:** antagonism, *Bacillus*, biocontrol, lettuce drop, *Sclerotinia minor* Jagger

## Abstract

Phytopathogenic *Sclerotinia minor* Jagger causes lettuce drop, a destructive soil-borne disease. As potential biocontrol agents for this disease, 2 of 31 bacterial strains isolated from soil samples from fields containing *S. minor* Jagger were identified using in vitro antagonistic assays against *S. minor* Jagger. Bioactivity experiments showed that Bac20 had higher inhibitory activity against *S. minor* Jagger than Bac45. Based on 16S rRNA sequences and phylogenetic analysis of a combination of sequences from *gyrA*, *rpoB*, *purH*, *polC*, and *groEL*, Bac20 and Bac45 were identified as *Bacillus velezensis* and *Bacillus subtilis*, respectively. Lipopeptide compounds produced by each strain were identified using matrix-assisted laser desorption ionization–time of flight mass spectrometry (MALDI–TOF MS) analysis. Both strains produced three types of lipopeptides, namely surfactins, iturins, and fengycins, whereas Bac20 showed the strongest intensity in its production of iturins, more than that of Bac45. Bac20 inhibited oxalic acid formation in early-stage lettuce leaves infected with *S. minor* Jagger, delaying pathogen infestation. Greenhouse experiments for controlling lettuce drop demonstrated that inoculation with Bac20 controlled lettuce drop by 71.7%. In conclusion, this study revealed that *B. velezensis* Bac20 has high potential for use as a biocontrol agent for controlling the lettuce drop caused by *S. minor* Jagger.

## 1. Introduction

Lettuce (*Lactuca sativa* L.) is a popular green leafy vegetable that plays an important role in daily diet and nutrition. It is widely grown in open fields, greenhouses, and controlled environmental agriculture facilities, making it available all year round [1]. Lettuce drop, caused by the fungal pathogens *Sclerotinia sclerotiorum* (Lib.) de Bary and *S. minor* Jagger, is a major threat to horticultural crops worldwide [2,3]. Although *S. sclerotiorum* (Lib.) de Bary is the predominant phytopathogen of lettuce in China, lettuce drop caused by *S. minor* Jagger has become increasingly severe in recent years [4]. *Sclerotinia minor* Jagger can infect more than 100 hosts, including lettuce, rapeseed, peanut, cabbage, and other economically important crops [5,6]. It overwinters as sclerotia in the soil or plant residues or on seeds. Sclerotia can survive in soil for many years. During the growing season, sclerotia in infected fields germinate by producing mycelia and occasionally ascospores to infect host plants [7,8].

*Sclerotinia minor* Jagger is a typically necrotrophic pathogen that encodes many pathogenicity factors, such as oxalic acid and cell wall-degrading enzymes, to break down the host’s defense system [9,10,11]. The genus *Sclerotinia*, which includes *S. sclerotiorum* (Lib.) de Bary, *S. rolfsii*, *S. trifoliorum*, and *S. minor* Jagger, produce the non-specific phytotoxin and key pathogenicity factor oxalic acid [12,13]. No plant genotypes are completely resistant to *S. minor* Jagger, making plant breeding for disease resistance difficult [2]. Chemical fungicides are widely used for disease control, but the control efficacy is limited because sclerotia are resistant to chemical fungicides, environmental stress, and microbial degradation [14,15]. In addition, extensive use of fungicides has raised public concerns over the environment and human health, meaning that it has become necessary to reduce the negative impact of fungicides.

Biological control using *Bacillus* spp. is a promising approach in plant protection [16,17]. *Bacillus* spp. produce abundant metabolites, such as lipopeptides (surfactin, fengycin, and bacillomycin), polyketides (macrolactin, bacillaene, and difficidin or oxidifficidin), and peptides (plantazolicin, amylocyclicin, and bacilysin), with broad-spectrum antifungal activity [18,19]. *Bacillus* spp. have been used as biological fungicides for the control of diseases, such as powdery mildew, gray mold, sheath blight, sclerotinia rot, and late blight. For example, *B. velezensis* AH2 has high inhibitory activity against *Botrytis cinerea*, *Pythium* spp., *Phytophthora* spp., *Sclerotinia* spp., *Penicillium* spp., and *Alternaria alternata* [20]. Additionally, *Bacillus* spp. colonize the host plant’s rhizosphere by forming biofilms, stimulating root system development, inducing systemic resistance, and improving abiotic stress tolerance [21,22]. Moreover, *Bacillus* spp. in plant rhizosphere have plant growth promoting abilities through biological nitrogen fixation, phosphate solubilization, siderophores production, and regulation of hormones [12,13,14].

Most studies on the biological control of *Sclerotinia* have focused on *S. sclerotiorum* (Lib.) de Bary, while *S. minor* Jagger has been less investigated. Furthermore, due to the complexity of the soil environment and the diversity of the soil microbiome, the control efficacy of biocontrol agents depends on their adaptability to the local soil microenvironment [23]. The aim of the present study, therefore, was to identify and characterize bacterial strains with inhibitory effects against *S. minor* Jagger from fields with continuous lettuce cultivation and to determine their efficacy for controlling lettuce drop.

## 2. Materials and Methods

### 2.1. Soil Sample Collection, Bacterial Isolation, and Screening

Nine soil samples were collected from the rhizosphere of lettuce plants in different fields in Shanghai, China. The lettuce cultivar ‘Yinong 3801’ was continuously grown in fields heavily infected by *S. minor* Jagger. After each soil sample was serially diluted with sterile distilled water, 100 µL aliquots of the soil suspension for each sample were spread homogenously onto a plate containing Luria–Bertani (LB) agar (5 g yeast extract, 10 g tryptone, 10 g NaCl, 15 g agar, pH 7.2). *Sclerotinia minor* Jagger SH09, a highly aggressive strain isolated from diseased lettuce in Shanghai, was grown in a conical flask with 150 mL liquid potato dextrose broth (PDB, potato dextrose agar (PDA) without agar) at 25 °C for 5 days. Mycelia were transferred to a glass grinder, and a homogenous mycelial fragment suspension was prepared.

After drying the plates with soil suspensions in a laminar flow hood, a 1.0 mL *S. minor* Jagger SH09 suspension containing mycelial fragments was spread onto the plates. After 24 h, bacterial colonies with a significant inhibition zone with SH09 were selected, and each colony was subsequently purified at least 3 times on LB plates. They were stored in 20% glycerol and deposited in the lab culture collection.

### 2.2. In Vitro Assessment of the Antagonistic Activity of Bacterial Strains Against S. minor Jagger

The antagonistic activity of the screened bacterial strains was assessed using the agar diffusion method [24]. A mycelial fragment suspension of *S. minor* Jagger SH09 was prepared, and 1.0 mL of fungal suspension was evenly spread on the plates containing PDA. After drying in a laminar flow hood, a sterile filter paper disc (5 mm) containing the bacterial cell suspension cultured in LB broth at 30 °C for 24 h at 180 rpm was placed at the center of one of the PDA plates. After the plates were incubated at 25 °C for 72 h, the inhibition zone diameter around the filter paper disc was measured. A paper disc with sterile water was used as a control, and three replicate plates per treatment were inoculated for each strain. The strains with the highest antagonistic activity were selected for further analysis.

### 2.3. Effect of Cell-Free Supernatants on the Hyphal Growth of S. minor Jagger

To evaluate the antagonistic activities of the cell-free supernatants (CFSs) from the selected bacterial strains against *S. minor* Jagger, a growth inhibition assay was carried out. To produce CFSs, the candidate strains were grown in LB broth incubated at 30 °C on a rotary shaker at 180 rpm for 72 h, centrifuged at 10,000 rpm for 10 min, and filtered through a 0.22 μm pore membrane (Millipore, Bedford, MA, USA). Sterilized melted PDA medium plates (9 mm in diameter) were prepared with 1, 2, 4, 6, and 8% CFS. Plates with sterile water instead of CFS were used as controls. To test the inhibitory efficacy, a mycelial plug (6 mm diameter) was cut from the edge of an *S. minor* Jagger SH09 colony cultured on PDA for 5 days and placed at the center of a PDA plate with CFS, and the plates were incubated at 25 °C. Each treatment was set up in triplicate. The inhibition of the mycelial growth was measured after 3 days of incubation, and the strains with high antagonistic activity were selected for further characterization.

### 2.4. Morphological and Molecular Identification of Bacterial Strains

To select the bacterial strains, they were cultured on LB plates at 30 °C for 24 h. Characteristics such as shape, color, size, regular or irregular, and convex or flat were observed. The 16S rRNA gene of each strain was amplified using primer pair 27F and 1492R [25], and purified PCR amplification products were submitted to Sangon Biotech Company Limited (Shanghai, China) for sequencing in both directions. The resulting sequences were evaluated using BLAST in the NCBI GenBank database for bacterial identification.

To distinguish closely related species at the same genus level, partial sequences of five housekeeping genes, namely gyrase subunit A (*gyrA*), RNA polymerase subunit B (*rpoB*), phosphoribosyl amino imidazolecarbox-amide formyltransferase (*purH*), DNA polymerase III subunit alpha (*polC*), and 60 kDa heat-shock protein groEL (*groEL*), were amplified using the primer pairs listed in Supplementary Table S1 [26]. A thermal cycling program was performed for 35 cycles after initial denaturation at 95 °C for 5 min, annealing between 45 and 55 °C for 30 s, and extension at 72 °C for 1.5 min. The amplified products were sent to Sangon Biotech Company Limited (Shanghai, China) for sequencing, and the resulting sequences were identified using BLAST in the GenBank database. A total of 12 sequences for the two strains were deposited in NCBI GenBank under accession numbers PP109156, PP102251, PP102252, PP102253, PP102254, PP102255, PP109157, PP102256, PP102257, PP102258, PP102259, and PP102260. Phylogenetic trees were constructed using the maximum likelihood (ML) method based on the combined five-gene dataset (*gyrA*-*rpoB*-*purH*-*polC*-*groEL* sequences) with 33 in-group taxa and 1 out-group taxa (*B. sonorensis*). The sequences of each gene were aligned using MAFFT v7.273 and then analyzed using Gblocks 0.91b to eliminate the ambiguously aligned positions and divergent regions before phylogenetic analyses. ML analyses were performed using RaxmlGUI v. 1.5, and ML bootstrap analysis of each ML tree was completed with a fast 1000 bootstrap frequency and the same parameter settings using the GTR + I + G model for nucleotide substitution [27,28]. A threshold ≥ 50% was used as the cut-off for significantly supported nodes.

### 2.5. Lipopeptide Detection Using Matrix-Assisted Laser Desorption Ionization–Time of Flight Mass Spectrometry (MALDI–TOF MS) Analysis

To identify the lipopeptides produced by the screened antagonistic strains, MALDI–TOF MS was employed [29]. The selected strains were incubated on LB plates at 30 °C for 24 h, and single colonies were selected and lysed in a centrifuge tube containing a matrix solution (cyano-4-hydroxycinnamic acid in 70% water, 30% acetonitrile, and 0.1% trifluoroacetic acid (*v*/*v*)). After the samples were homogenized and centrifuged at 5000 rpm, 1.0 μL of the sample solution was spotted onto a 384-target disk for ultraflextreme MALDI–TOF mass spectrometer (Bruker Daltonics, Billerica, MA, USA) and allowed to dry before analysis. MALDI–TOF MS was performed using an ultraflextreme™ MALDI–TOF device with a smart beam laser (Bruker, Bremen, Germany). Measurements were performed in reflection mode and at an ion source acceleration voltage of 20 kV. The mass spectra were stored in a low mass range region between 0.1 and 2 kD.

### 2.6. Biocontrol Efficacy of Selected Bacterial Strains

Fresh lettuce leaves of cultivar ‘Yinong 3801’ with similar size were rinsed with sterile water and dried, and the leaves were placed in a Petri dish with moist filter paper at the bottom. Bacterial candidates grown in LB medium were diluted 1 × 10^7^ CFU/mL and then sprayed onto the surface of the lettuce leaves. Each leaf was sprayed with 2 mL of bacterial suspension, and sterile water was used as a negative control. After lettuce leaves were dried, a hyphal disc (0.6 mm diameter) from an *S. minor* Jagger colony was placed on each leaf. After culturing for 2 days at 25 °C, disease symptoms were observed on lettuce leaves. The experiment was conducted three times with five leaves per treatment. Disease severity was assessed on each plant using a scale of 0–4 [30], where 0 = healthy leaf; 1 = lesions less than 1.5 cm on leaf; 2 = lesions less than 3.5 cm on leaf; 3 = lesions less than 5.5 cm on leaf; 4 = lesions greater than 5.5 cm or most leaves rotten. Disease measurements included the following: disease incidence (%) = (number of diseased leaves/total number of leaves investigated) × 100; disease severity = Σ (disease ratings × number of diseased leaves)/(maximum rating value × total number of leaves) × 100; and biological control efficacy (%) = (disease severity index of control − disease severity index of treatment/disease severity index of control) × 100.

### 2.7. Detection of Oxalic Acid Accumulation in Detached Leaves

To determine whether candidate strains could inhibit oxalic acid production in *S. minor* Jagger, an assay was set up using lettuce leaves. A 2 mL bacterial cell suspension of each candidate strain at a concentration of 1 × 10^7^ CFU/mL was prepared and sprayed on the surface of each freshly detached leaf, and the leaves were placed at the bottom of a container with wet filter paper. Sterile water was used as a control, and each treatment was set up in triplicate. A disc (5 mm diameter) from the margin of a 3-day-old colony of an *S. minor* Jagger isolate was placed onto the middle of each leaf. The container was incubated at 25 °C under an alternating cycle of 12 h light and 12 h dark for 2 days. The parts of leaves with disease symptoms were stained with 0.01% bromophenol blue, and color changes were observed on each leaf.

### 2.8. Determination of the Ability to Inhibit Oxalic Acid Production

To further examine the inhibitory effects on oxalic acid, an assay was conducted in a liquid medium. First, bacterial CFS for each strain was prepared, and then 100 mL yeast extract, peptone and dextrose (YEPD) medium (1% peptone, 2% dextrose, with 0.04 g adenine per liter) in a triangular flask was amended with CFS at final concentrations of 0, 2, 4, 6, and 8%. A hyphal disc from *S. minor* Jagger SH09 was added, and flasks were incubated at 25 °C on an orbital shaker (180 rpm). After incubation for 72 h, the YEPD medium was centrifuged at 12,000 rpm for 10 min, and supernatants were centrifuged at 12,000 rpm for another 10 min. The oxalic acid concentration in the supernatant was determined using a colorimetric method [31,32]. To generate the standard curve for oxalate, a series of sodium oxalate concentrations (0, 2, 4, 6, and 8%) at 2 mg/mL were added to a buffer containing 2 mL of 0.5 mg/mL FeCl_3_ solution, 20 mL HCl-KCl buffer (KCl 50 mM, pH = 2), and 1.2 mL of 5 mg/mL sulfosalicylic acid solution in a 50 mL flask. The final volume was adjusted to 25 mL by adding double-distilled water. The flask was swirled and incubated at 25 °C for 30 min [32]. Absorbance was measured with a spectrophotometer UV–vis (Thermo Scientific-Genesys 150, Milan, Italy) at 510 nm. The experiments were performed in triplicate and independently repeated twice.

### 2.9. Biocontrol Assay in Pot Experiments in the Greenhouse

The effects of the selected bacterial strains against lettuce drop caused by *S. minor* Jagger were determined. Lettuce seeds were sterilized with a 2% sodium hypochlorite solution and sown in a plastic tray containing autoclaved mixing soil (peat:vermiculite:farm yard soil in a 2:1:1 ratio) in an illumination incubator. After 2 weeks, each seedling was transplanted into a plastic pot (14 cm diameter), and the pots were moved to a greenhouse with a relative humidity of 80–90% and a temperature of 20–25 °C. After 25 days, lettuce seedlings of similar sizes were selected, and 20 mature sclerotia of *S. minor* Jagger growing on PDA were placed into leaf axils near the soil. A 100 mL bacterial cell suspension (about 1 × 10^7^ CFU/mL) was applied to each seedling. Twenty pots were used for each treatment, and each treatment had three independent replications. The treatment with sterile water was used as a control. After incubation for 10 days, the bacterial cell suspension was applied again. Disease incidence, disease severity, and control efficacy were assessed 25 days after inoculation. Disease severity was assessed for each plant using a 0–4 scale [33], where 0 = healthy plant; 1 = 1–4 leaves around the lower part of the plant that have turned brown and soft; 2 = 5–10 leaves around the lower part of the plant that have turned brown and soft, and with disease spots that are extended to the upper part of the main root; 3 = wilt appearing on the leaves of the whole plant; 4 = plant has collapsed or died.

### 2.10. Plant Growth Promotion of Lettuce Seedlings Caused by Biocontrol Agent B. velezensis

To evaluate the plant growth promotion of lettuce seedlings caused by the biocontrol agent, plant growth indexes, namely the number of leaves, seedling height, root length, fresh weight, and dry weight, were examined. The 20 mL cell suspension (about 1 × 10^7^ CFU/mL) produced by the Bac20 was used to inoculate each individual plant 3 and 10 days after transplant, for bacterial effective colonization. The control group was inoculated with the same amount of sterile water. Each treatment was repeated with 15 plants. Plant growth indexes were measured after cultivation for 20 days in the greenhouse.

All statistical analyses were performed using SPSS 26.0 (IBM Corp., Armonk, NY, USA), and the level of significance for the LSD test was set at *p* < 0.05.

## 3. Results

### 3.1. Isolation and Screening of Bacterial Strains

**Figure 1:**
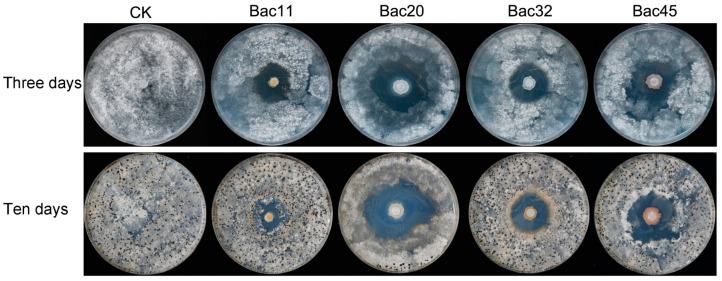
Antifungal activity of four bacterial strains (Bac11, Bac20, Bac32 and Bac45) against hyphae growth of *Sclerotinia minor* Jagger SH09 after inoculation for 3 days and 10 days.

A total of 31 bacterial colonies with obvious fungal inhibition zones were isolated from the 9 soil samples and further screened for antagonistic activity in an in vitro assay. Among the strains, four showing large inhibition zones (diameter of more than 25.0 mm) were selected and designated Bac11, Bac20, Bac32, and Bac45 (Figure 1). After 3 days, strain Bac20 produced the largest inhibition zone (45.44 ± 0.94 mm), followed by Bac45 (38.22 ± 1.15 mm), Bac11 (30.33 ± 0.47 mm), and Bac32 (29.67 ± 0.47 mm). Although numerous sclerotia were produced by *S. minor* Jagger on the plates inoculated with strains Bac11, Bac32, and Bac45, only a few sclerotia were distributed at the margin of plates inoculated with Bac20 after incubation for 10 days (Figure 1). This demonstrated that strain Bac20 inhibited sclerotium formation. Based on the inhibition zone diameters, two strains, Bac20 and Bac45, were selected for further study.

### 3.2. Antagonistic Assay of Cell-Free Supernatants

**Figure 2:**
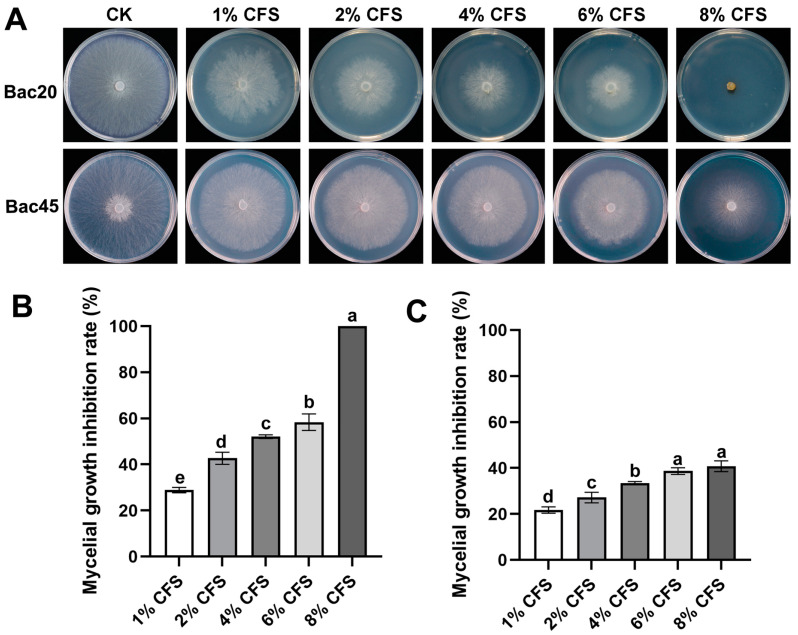
Antifungal activity of cell-free supernatants (CFS) produced by bacterial strains against hyphae growth of *Sclerotinia minor* Jagger SH09 after incubation for 72 h. (**A**) Effect of different concentrations of CFS produced by strain Bac20 or Bac45 on *S. minor* Jagger. (**B**,**C**) Statistical analysis results for CFS inhibiting mycelial growth of *S. minor* Jagger Bac20 (**B**) and Bac45 (**C**). Vertical bars represent standard errors of the means. Different letters indicate significant difference among different treatments (n = 3, *p* < 0.05).

An in vitro antagonistic assay showed that the CFS dilutions of Bac20 had the highest inhibitory activity against the colony growth of *S. minor* Jagger SH09 after incubation for 72 h (Figure 2). Specifically, colony growth was completely inhibited on PDA plates containing 8% CFS produced by Bac20. Both strains exhibited the highest inhibition at 8% CFS (100% for Bac20 and 40.8% for Bac45) (Figure 2).

### 3.3. Identification of the Selected Bacterial Strains

On LB agar plates, the Bac20 colonies were yellowish and round with irregular colony edges, while the Bac45 colonies were round, milky, rough, yellowish or translucent, and wrinkled. The Gram reaction showed that both strains were Gram positive. The BLAST search analysis of the 16S rRNA gene showed that Bac20 had 99% identity with the *B. velezensis* strain M6 (GenBank No. MK226560.1) and that Bac45 had 99.86% identity with *B. subtilis* PSB8 (GenBank No. OQ955764.1).

**Figure 3:**
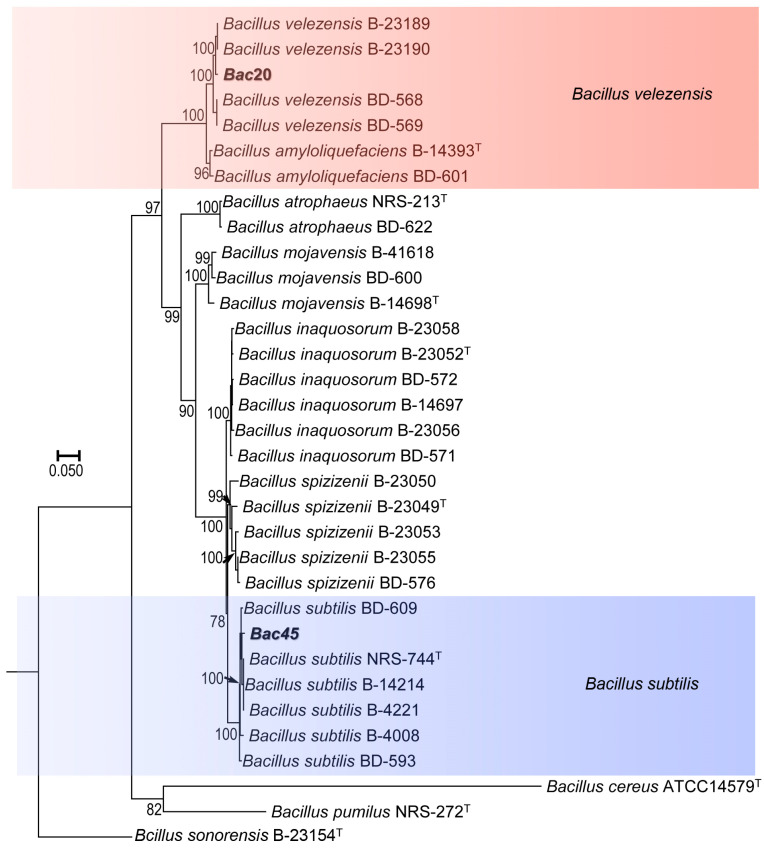
Maximum likelihood (ML) tree generated from the combined *gyrA*, *rpoB*, *purH*, *polC* and *groEL* gene sequences of 33 taxa of *Bacillus*. The tree is rooted with *B. sonorensis*. The type strains isolated from the rhizosphere of lettuce plants are shown in bold.

To delineate closely related species boundaries in *Bacillus*, a phylogenetic tree was constructed using a sequence combination of five genes (*gyrA*, *rpoB*, *purH*, *polC*, and *groEL*). The phylogenetic tree showed that all studied isolates were separated into different clades (Figure 3). The relationship of almost all reference isolates was clearly distinguished at the species level. Bac20 clustered with *B. velezensis* B-23189, B-23190, BD-568, and BD-569, as well as *Bacillus amyloliquefaciens* B-14393^T^ and BD-601, as a clade with 100% ML bootstrap branch support. Therefore, strain Bac20 was identified as *B. velezensis*. Similarly, strain Bac45 clustered with *B. subtilis* BD-609, NRS-744^T^, B-14214, B-4221/B-4008, and BD-539, as a distinct clade with high ML (100%) bootstrap branch support, and was identified as *B. subtilis*.

### 3.4. Lipopeptide Detection by MALDI–TOF MS Analyses

**Figure 4:**
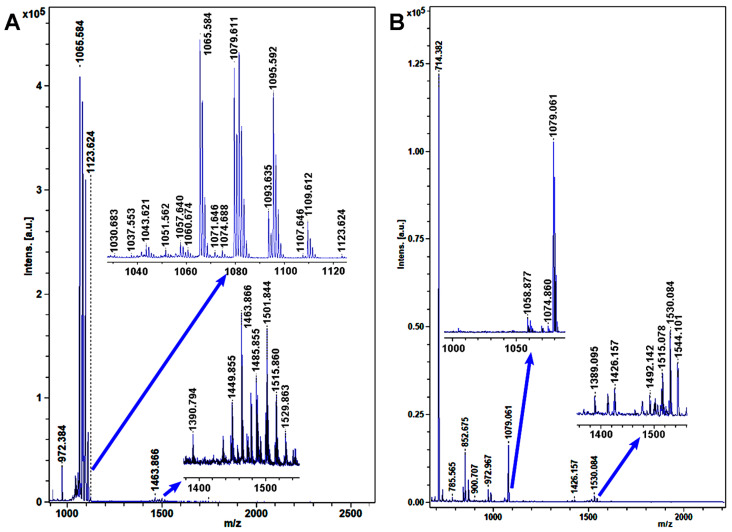
Matrix-assisted laser desorption ionization–time of flight mass spectrometry (MALDI–TOF MS) analysis of lipopeptide compounds produced by two bacterial strains. (**A**) Bac20. (**B**) Bac45.

MALDI–TOF MS was carried out to detect and identify lipopeptide compounds from Bac20 and Bac45. Multiple peaks of lipopeptide compounds produced by Bac20 and Bac45 indicated the presence of surfactins, iturins, and fengycins, although the intensity of the peaks was much higher in Bac20 (Figure 4). In strain Bac20, the strong major peaks observed revealed the presence of iturin A (*m*/*z* 1065.584, 1079.611, 1095.592) [34]. The peaks with a lower intensity in the mass range of *m*/*z* = 1450–1550 were typical of fengycins [34]. In strain Bac45, the major peak exhibited the presence of iturin (*m*/*z* 1079.611), while peaks with a low intensity exhibited the mass spectrum of fengycins (*m*/*z* 1515.078, 1530.084, 1544.101) [34,35]. In addition, several peaks were observed in both strains, probably related to surfactin production. All mass peaks and the corresponding lipopeptides are listed in Table 1.

### 3.5. Effect of Bac20 and Bac45 Against Lettuce Drop on Detached Leaves

**Figure 5:**
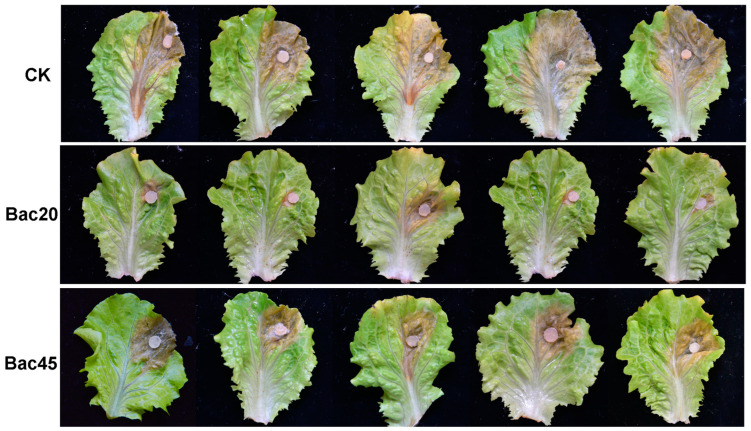
Symptoms of disease on detached leaves of lettuce after inoculation for 3 days. Top part: control (co-inoculation by sterile water + *Sclerotinia minor* Jagger SH09). Middle part: co-inoculation by Bac20 + *S. minor* Jagger SH09. Bottom part: Bac45 + *S. minor* Jagger SH09.

To compare the potential biocontrol efficacy of Bac20 and Bac45 against the lettuce drop caused by *S. minor* Jagger SH09, an assay was carried out on detached leaves. Brown lesions of different sizes appeared around the fungal inoculation discs on the leaves of different treatments 3 days after inoculation. Co-inoculation with Bac20 and Bac45 significantly reduced lesion sizes compared with the control (Figure 5), but Bac20 showed much higher efficacy. Statistical analysis showed that co-inoculation with Bac20 and Bac45 significantly reduced both the disease incidence and severity (Table 2). However, significant differences were shown between the two strains (*p* < 0.05). Co-inoculation of Bac20 + *S. minor* Jagger SH09 resulted in a much higher control efficacy (75.79%) and decreased the incidence and disease severity by 29.63% and 65.74%, respectively.

### 3.6. Oxalic Acid Formation on Detached Leaves and the Ability to Inhibit Oxalic Acid Production

**Figure 6:**
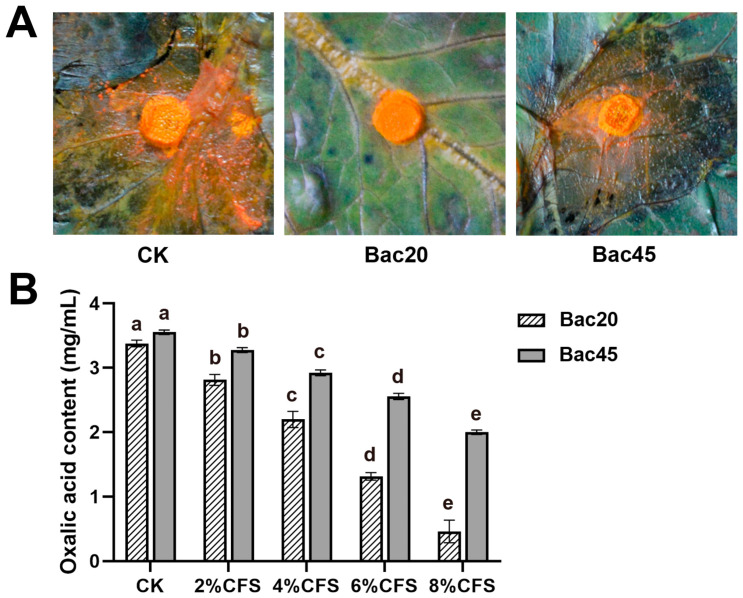
Candidate strains inhibited oxalic acid synthesis by *Sclerotinia minor* Jagger. (**A**) Detecting *S. minor* Jagger oxalic acid formation by bromophenol blue staining on lettuce leaves. The leaf color changed from blue to bright yellow, indicating the formation of oxalic acid. (**B**) Inhibitory effects of Bac20 and Bac45 on oxalic acid synthesis by *S. minor* Jagger with different concentrations of CFS. Bars are representative of the mean, and error bars are representative of the standard deviation of three independent experiments. Different letters indicate significant difference among different treatments (n = 3, *p* < 0.05).

The bromophenol blue leaf staining results indicate that treatment with cell suspensions of Bac20 and Bac45 cultured for 2 days reduced oxalic acid accumulation on the lesions around the hyphae discs compared with the control (Figure 6). As assessed by color changes, abundant oxalic acid accumulation was observed in all treatments, but the changes varied markedly among the treatments. Using the CFS from Bac20, slight color changes were observed around hyphae discs (Figure 6), demonstrating less oxalic acid accumulation. Partially bright yellow color around a hyphal disc was observed with the CFS from Bac45, but there was less color than in the control, indicating relatively more oxalic acid accumulation. This illustrates that both strains inhibited oxalic acid production, but that stronger inhibition was observed when using Bac20 CFS. The ability of the CFS of Bac20 and Bac45 to inhibit oxalic acid production from *S. minor* Jagger was determined. Both strains effectively inhibited oxalic acid production (Figure 6). In strain Bac20, the highest inhibitory efficacy of the CFS was 86.4% at an 8% concentration, while in Bac45, the highest inhibitory efficacy was 43.8% at an 8% concentration. This further confirms that the CFS from strain Bac20 had much stronger inhibitory activity against oxalic acid production.

### 3.7. Biocontrol Effect of Bacterial Strains Under Greenhouse Conditions

**Figure 7:**
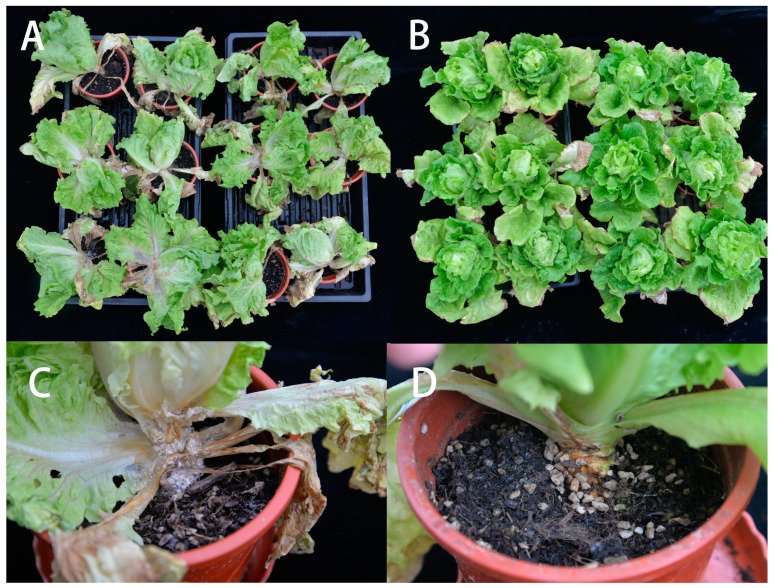
Effect of inoculation with *Bacillus velezensis* Bac20 on lettuce drop for 25 days after inoculation. (**A**) Inoculation with pathogen *Sclerotinia minor* Jagger SH09 (control). (**B**) Co-inoculation with strains *S. minor* Jagger SH09 + Bac20. (**C**,**D**) Close up view of disease symptoms. (**C**) Symptom of crown rot in (**A**). (**D**) The initial symptom on a root crown in (**B**).

Based on the previous results, strain Bac20 was selected for in vivo greenhouse experiments. Inoculation tests showed that Bac20 delayed symptoms and significantly reduced disease incidence and severity. Foliage symptoms were observed 15 days after inoculation in control plants inoculated with *S. minor* Jagger SH09 sclerotia, but there were no symptoms in plants co-inoculated with Bac20. Symptoms were more severe after 20 days but still less severe on leaves co-inoculated with Bac20. In plants inoculated with only *S. minor* Jagger SH09, pathogen infection caused brown lesions on leaves and crown rot, and white fluffy mycelia were observed on infected tissues. Obvious leaf symptoms were observed 25 days after inoculation on plants co-inoculated with Bac20 + SH09. Although the lesions were observed on basal parts of the stem, no typical symptoms of crown rot were found (Figure 7). Sudden wilting of leaf crown rot with abundant white mycelia and black sclerotia occurred in control plants inoculated with only SH09 (Figure 7). Statistical analysis showed that co-inoculation with Bac20 + SH09 resulted in the greatest control efficacy (71.7%) (Table 3) and decreased the disease incidence and severity by 22.1% and 57.8%, respectively, 25 days after inoculation (*p* < 0.05).

### 3.8. Growth-Promoting Effect of B. velezensis on Lettuce

**Figure 8:**
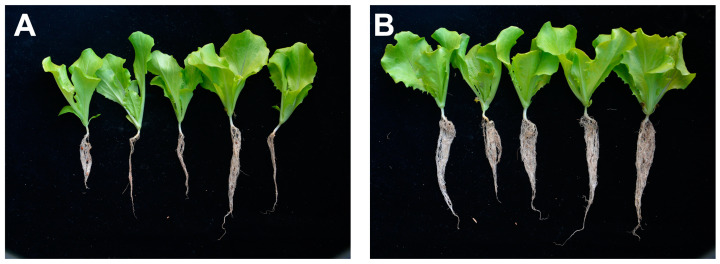
Efficacy of lettuce plant growth promotion when inoculated with *B. velezensis* Bac20. (**A**) Inoculation with sterile water; (**B**) inoculation with *B. velezensis* Bac20.

The effect of strain Bac20 on lettuce biomass during the seedling stage is shown in Table 4. To evaluate the effect of growth promotion, the number of leaves, plant height, root length, fresh weight, and dry weight of lettuce seedlings were determined at 20 days after *B. velezensis* Bac20 treatment. The strain significantly increased the root length, fresh weight, and dry weight of lettuce seedlings (Figure 8). However, the leaf number and plant height were not different among treatments. The results indicate that *B. velezensis* promoted root system growth in lettuce plants, thereby increasing lettuce biomass.

## 4. Discussion

The present study identified two indigenous *Bacillus* strains, *B. velezensis* Bac20 and *B. subtilis* Bac45, with high inhibitory activity against the hyphal growth of *S. minor* Jagger, with Bac20 showing much higher inhibition. In vitro assays showed that cell-free supernatants (CFS) from Bac20 had 100% inhibitory activity against the hyphal growth of *S. minor* Jagger when compared with Bac45, which showed 58.4% inhibition at an 8% CFS concentration. A detached leaf assay showed that co-inoculation with Bac20 + *S. minor* Jagger reduced lesion sizes by 76%, demonstrating that Bac20 has potential as an effective biocontrol agent. This difference could potentially involve antimicrobial compounds.

Antimicrobial lipopeptides produced by *Bacillus* spp. have been investigated for their biological activity in suppressing disease [38]. These strains produced three families of lipopeptide antibiotics, namely surfactins, iturins, and fengycins, according to the structure of the cyclic peptides [39]. In this study, both Bac20 and Bac45 produced three families of lipopeptide compounds, but variants of these compounds in each family and the corresponding peak intensity differed. Surfactin can enter cell bilayers as an antibacterial agent, and it chelates cations, solubilizes membranes, and lyses pathogens by pore creation [40,41]. The antifungal action of iturin works via reactive oxygen species (ROS) accumulation, Hog1 mitogen-activated protein kinase (MAPK) activation, and cell wall integrity defects [42]. Fengycins have been reported for their anti-fungal activity by perturbing the membrane and inducing apoptosis at low concentrations and necrosis at high concentrations [43,44]. MALDI–TOF MS analysis demonstrated that the peaks assigned to surfactins and fengycins were similar in both strains, while the peaks with low intensity differed in both strains (C_14_–C_15_), possibly due to differences in bioactivity between strains. Previous studies have shown that iturin and fengycin are promising antifungal compounds [39]. Iturins exhibit strong antifungal activity against a wide range of fungi [39]. Although fengycins show strong fungitoxic activity, specifically against filamentous fungi [39], and fengycin A and B are involved in the antifungal activity of *B. subtilis* against *S. sclerotiorum* (Lib.) de Bary, their role may have been limited by their relatively low intensity in this study. In contrast, surfactins have little or no marked antifungal activities [45,46]. The iturin variants may be related to the high antifungal activity in strain Bac20. However, *Bacillus thuringiensis* (Bt) is a unique bacterium, which has been known for a biological pesticide. Most strains of Bt have *cry* genes encoding δ-endotoxin insecticidal proteins, which are present on the plasmids [47]. Thus, it is widely used as a biopesticide to control pests in agriculture for substitution of chemical pesticides [48,49].

In addition, in vivo and in vitro assays showed that both Bac20 and Bac45 inhibited oxalic acid production in *S. minor* Jagger (Figure 6), but Bac20 had a much higher inhibitory activity. Oxalic acid secreted by *S. minor* Jagger meets with a lower environmental pH, providing a favorable environment for the lytic activities of cell wall-degrading enzymes and chelating calcium in host cell walls, making the plants more susceptible to infection by *S. minor* Jagger [50,51]. Calcium oxalate can also act as a signaling molecule to induce programed cell death and to manipulate oxidative reduction in plants, disrupting the host defense system [9,52]. These results provide novel information for understanding the biocontrol mechanisms of *Bacillus* spp. against *S. minor* Jagger. However, although oxalic acid production was inhibited, the accurate mechanism of sclerotium antagonism still needs to be analyzed.

The antagonistic activity of Bac20 was further confirmed in greenhouse pot experiments, which showed a remarkable decline in the severity of lettuce drop symptoms on lettuce leaves. Bac20 showed 71% efficacy toward the reduction of *S. minor* Jagger in an in vivo assay and lowered the disease incidence and severity in lettuce plants. *B. velezensis* has been reported as a powerful biocontrol agent for many plant diseases, such as grape white rot [53], pepper and tomato gray mold [54,55,56], sweet potato wilt, and black rot diseases [56]. In addition, this strain also confirmed its ability to promote lettuce seedling growth, which is consistent with previous findings for *B. velezensis* strain BAC03 [57]. It could produce indole-3-acetic acid and ammonia to enhance the growth of nine different plant species. The evaluation of plant growth-promoting *B. velezensis* strain XT1 indicated that an effect due to an indirect interaction with native soil or root microorganisms should be considered [58]. Plant-growth-promoting *Bacillus* spp. can positively impact the native rhizosphere microbial communities [59], suggesting the significance of the rhizosphere microbiome for plant and soil health. Therefore, *B. velezensis* showed strong ability of disease control and combined with plant growth promotion, demonstrating its great value for plant protection.

## 5. Conclusions

Overall, our study showed that the novel strain Bac20, identified as *B. velezensis,* not only had much better control efficacy on lettuce drop but also promoted lettuce seedling growth. The strain produced strong antifungal lipopeptides and was determined to be a potent biocontrol agent against the lettuce drop caused by *S. minor* Jagger. The use of this strain decreased the incidence of lettuce drop and suppressed the disease under greenhouse conditions. Therefore, *B. velezensis* Bac20, bearing considerable and practical significance, can be employed to develop a powerful biocontrol agent for lettuce drop management. This will provide a major impetus for the more widespread and comprehensive use of plant protection bioproducts in agriculture.

## Figures and Tables

**Table 1:** Main mass peaks of the lipopeptides produced from *Bacillus velezensis* Bac20 and *B. subtilis* Bac45 by MALDI–TOF MS.

Species	Mass Peak (*m*/*z*)	Family	Assignment	References
*Bacillus velezensis* Bac20	1030.683	C13 surfactin	[M + Na]^+^	[36]
	1060.674	C14 surfactin	[M + K]^+^	[34]
	1074.688	C15 surfactin	[M + K]^+^	[34]
	1057.640	C15 iturin	[M + H]^+^	[34]
	**1065.584**	C14 iturin A	[M + Na]^+^	[34]
	**1079.611**	C15 iturin A	[M + Na]^+^	[34]
	**1095.592**	C15 iturin A	[M + K]^+^	[34]
	1449.855	C15 fengycin	[M + H]^+^	[37]
	**1463.855**	C16 fengycin A	[M + H]^+^	[34]
	**1485.855**	C16 fengycin A	[M + Na]^+^	[34]
	**1501.844**	C16 fengycin A	[M + K]^+^	[34]
	1515.860	C17 fengycin A	[M + K]^+^	[34]
	1529.863	C16 fengycin B	[M + K]^+^	[34]
*B. subtilis* Bac45	1030.683	C13 surfactin	[M + Na]^+^	[36]
	1058.877	C15 surfactin	[M + Na]^+^	[36]
	1074.860	C15 surfactin	[M + K]^+^	[34]
	1060.674	C14 surfactin	[M + K]^+^	[34]
	1057.640	C15 iturin	[M + H]^+^	[34]
	1065.584	C14 iturin A	[M + Na]^+^	[34]
	**1079.611**	C15 iturin	[M + H]^+^	[34]
	1095.592	C15 iturin A	[M + K]^+^	[34]
	**1515.078**	C17 fengycin A	[M + K]^+^	[34]
	**1530.084**	C16 fengycin B	[M + K]^+^	[34]
	**1544.101**	C17 fengycin B	[M + K]^+^	[35]

**Table 2:** Inhibitory efficacy of two antagonistic bacterial strains against lettuce drop after inoculation for 3 days on detached leaves.

Inoculated Strains	Disease Incidence (%)	Disease Severity	Control Efficacy (%)
*Sclerotinia minor* Jagger + Bac20	70.37 ± 3.70 b	21.29 ± 1.61 c	75.79
*S. minor* Jagger + Bac45	100 a	50.93 ± 0.93 b	41.46
Sterile water + *S. minor* Jagger	100 a	87.03 ± 0.93 a	-

Different letters indicate significant difference among different treatments (n = 3, *p* < 0.05).

**Table 3:** Effects of antagonistic bacterial strain Bac20 in controlling lettuce sclerotinia after 25 days of inoculation in greenhouse.

Inoculated Strains	Disease Incidence (%)	Disease Severity	Control Efficacy (%)
*Sclerotinia minor* Jagger + Bac20	66.67 ± 3.76 b	22.78 ± 2.22 b	71.7
*S. minor* Jagger	88.78 ± 2.11 a	80.55 ± 0.55 a	-

Different letters indicate significant difference among different treatments (n = 3, *p* < 0.05).

**Table 4:** Effect of *B. velezensis* Bac20 on lettuce growth and biomass.

Treatment	Biomass of Lettuce				
	Number of Leaves	Seedling Length (cm)	Root Length (cm)	Fresh Weight (g)	Dry Weight (g)
Control	6.33 ± 0.52 a	8.53 ± 0.45 a	8.33 ± 1.02 b	2.00 ± 0.66 b	0.12 ± 0.01 b
Bac20	6.67 ± 0.82 a	9.03 ± 0.29 a	11.48 ± 1.16 a	3.50 ± 0.44 a	0.23 ± 0.04 a

Means in the columns with different letters are significantly different according to LSD tests (n = 15, *p* < 0.05).

## Data Availability

Data are contained within the article and Supplementary Materials. Sequences for the two strains were deposited in NCBI GenBank.

## Supplementary Materials

The following supporting information can be downloaded at https://www.mdpi.com/article/10.3390/microorganisms13010068/s1, Table S1: The primer sequences used in this study.
