# Supplementary material for: Biocontrol Potential of Rhizospheric Bacillus Strains Against Sclerotinia minor Jagger Causing Lettuce Drop
Source: Microorganisms. 2025 Jan 2;13(1):68. doi: 10.3390/microorganisms13010068 (PMC11767259; doi:10.3390/microorganisms13010068)
Supplement: Supplementary file 1 [file microorganisms-13-00068-s001.zip › microorganisms-3368955-supplementary.pdf]

Table S1. The primer sequences used in this study.

Gene	Primers	Sequence (5’-3’)
16S rRNA	16S-27F	AGAGTTTGATCCTGGCTCAG
	16S-1492R	AAGGAGGTGATCCAGCCGCA
gyrA	*gyrA*-42F	CAGTCAGGAAATGCGTACGTCCTT
	gyrA-1066R	CAAGGTAATGCTCCAGGCATTGCT
rpoB	*rpoB*-2292R	GACGTGGGATGGCTACAACT
	rpoB-3354R	ATTGTCGCCTTTAACGATGG
purH	*purH*-70F	ACAGAGCTTGGCGTTGAAGT
	purH-101R	GCTTCTTGGCTGAATGAAGG
polC	*polC*-1505F	TTGTCGCTCAYAATGCAAGC
	polC-2337R	YTCAAGCATTTCRTCTGTCG
groEL	*groEL*-550F	GAGCTTGAAGTKGTTGAAGG
	groEL-1497R	TGAGCGTGTWACTTTTGTWG
